# Development of Near-Infrared Models for Selenium Content in the Pacific Oyster (*Crassostrea gigas*)

**DOI:** 10.3390/foods15020365

**Published:** 2026-01-20

**Authors:** Yousen Zhang, Lehai Ni, Yuting Meng, Cuiju Cui, Qihao Luo, Zan Li, Guohua Sun, Yanwei Feng, Xiaohui Xu, Jianmin Yang, Weijun Wang

**Affiliations:** 1 School of Fisheries, Ludong University, Yantai 264025, China; 19909024486@163.com (Y.Z.); cuicuiju@163.com (C.C.); lizanlxm@163.com (Z.L.); sgh_smile@163.com (G.S.); fywzxm1228@163.com (Y.F.); xxh83121@163.com (X.X.); ladderup@126.com (J.Y.); 2 General Station of Fishery Development and Resource Conservation in Shandong Province, Jinan 250013, China; nilehai@163.com; 3 College of Fisheries and Life Science, Shanghai Ocean University, Shanghai 201306, China; mengyuting0611@163.com; 4 Yantai Kongtong Island Industrial Co., Ltd., Yantai 264000, China; 17854266371@163.com

**Keywords:** NIR spectroscopy, selenium content, *Crassostrea gigas*, quantitative analysis

## Abstract

Near-infrared (NIR) spectroscopy is a vital non-destructive analytical tool in the food and aquaculture industries. This study pioneers the application of portable NIR spectrometers for evaluating selenium (Se) content in the Pacific oyster (*Crassostrea gigas*). We developed quantitative and qualitative models to predict selenium levels in oyster tissue, representing a novel application for monitoring trace elements in marine organisms. Quantitative models were developed using partial least squares (PLS) regression on spectra collected with two portable spectrometers (Micro NIR 1700, Micro PHAZIR RX) and a benchtop FT-NIR instrument, with validation via cross-validation and an independent set. Qualitative models were also constructed to categorize Se content into three levels: 0–1, 1–3, and >3 mg/kg. For quantitative analysis, the Micro NIR 1700 model performed robustly in external validation (RP = 0.932; RMSEP = 0.392; RPD = 2.46). The Micro PHAZIR RX model achieved the highest RC (0.988) and the lowest RMSEC (0.233), yet cross-validation indicated a potential risk of overfitting. In contrast, the FT-NIR instrument yielded the best external predictive ability for powdered samples (RP = 0.954, RPD = 2.60), highlighting its high precision under laboratory conditions. For qualitative discrimination, the Micro PHAZIR RX’s classification module achieved a 100% correct recognition rate (AUC = 0.937). The models based on the Micro NIR 1700 and FT-NIR instruments showed cumulative contribution rates (CCR) of 98.61% and 97.59%, respectively, with high performance indices (PI) of 89.3 and 90.2, confirming their effective discrimination capability. The models established in this study enable the rapid, on-site detection of Se content in oyster samples, underscoring the significant potential of portable NIR spectroscopy for selenium analysis in shellfish.

## 1. Introduction

Selenium (Se) is an essential trace element with a narrow margin between nutritional benefit and toxicity, meaning its biological effects in humans and animals are fundamentally dose-dependent [1,2]. It plays critical roles in processes like antioxidant defense and immune regulation, primarily by modulating selenoproteins such as glutathione peroxidase (GPx) to maintain redox homeostasis and support health [3,4,5]. Consequently, both deficiency and excessive intake can be detrimental, highlighting the critical importance of monitoring and maintaining selenium levels within a safe and optimal physiological range.

The Pacific oyster (*Crassostrea gigas*) is the most representative farmed shellfish species along the coast of China. According to industry projections, oyster aquaculture production in China is projected to reach approximately 7.2 million tons by 2025, representing a critical component of the aquaculture industry that provides substantial economic value [6]. It is not only highly nutritious and rich in protein but, as a filter-feeding organism, also exhibits high feeding rates and a strong capacity to accumulate trace elements from the environment [7]. The selenium content in its tissues can effectively reflect changes in the aquatic environment, making it an ideal bio-indicator for monitoring exposure levels of heavy metals and metalloids in marine areas [8,9]. The selenium present in oysters is predominantly in the organic form, bound to proteins, representing a high-quality natural selenium source for human nutrition [10]. Consequently, the Pacific oyster serves as a typical model shellfish for investigating the detection and characterization of selenium in aquatic foods.

The determination of selenium in aquatic products currently relies on traditional analytical techniques, primarily atomic absorption spectroscopy (AAS) and inductively coupled plasma mass spectrometry (ICP-MS) [11]. While these methods provide high sensitivity and accuracy, they suffer from several drawbacks: complex sample preparation, time-consuming procedures, high cost, and typically destructive sample processing through acid digestion [12,13,14]. These limitations not only compromise sample integrity and in situ characteristics but also render the techniques unsuitable for the high-throughput, rapid detection demands of modern aquaculture. Consequently, there is a pressing need for the development of rapid, non-destructive analytical techniques in this field.

Near-infrared (NIR) spectroscopy is a non-destructive analytical technique based on the absorption arising from the overtones and combinations of fundamental vibrations of bonds such as C–H, N–H, and O–H [15]. It offers significant advantages, including rapid analysis, being environmentally friendly (green), and requiring no chemical reagents [16]. NIR spectroscopy has been widely applied to aquatic products, such as fish and bivalves, for rapid assessment of quality attributes including moisture, protein, lipid content, and freshness, demonstrating its effectiveness for routine and non-destructive analysis [17,18]. The sensitivity of NIR spectra to molecular structures associated with organically bound elements (e.g., selenium) further supports its potential for quality and safety monitoring in food and aquaculture systems [19,20]. However, the quantitative determination of specific trace elements such as selenium in complex matrices like shellfish remains challenging due to their low concentrations and indirect spectral responses [21].

Although elemental selenium itself generally lacks distinct characteristic absorption in the NIR region, its incorporation into proteins in the form of Selenoamino acids (e.g., selenocysteine, Sec) alters the local chemical environment [22]. This modification, in turn, can induce measurable shifts in the vibrational frequency or absorption intensity of adjacent bonds such as N–H, O–H, and C–N, a phenomenon supported by studies on selenium-containing amino acids using vibrational spectroscopy [23,24].

Fourier transform near-infrared (FT-NIR) spectroscopy offers high spectral resolution and superior signal-to-noise ratio, making it well-suited for precise laboratory-based quantitative analysis [25]. In contrast, portable spectrometers (e.g., Micro NIR 1700) and handheld spectrometers (e.g., Micro PHAZIR RX) feature compact structures, user-friendly operation, and excellent field adaptability, enabling immediate on-site testing directly in processing plants or at sample collection sites [26,27]. The distinct spectral acquisition mechanisms, wavelength coverage, and resolutions of these different types of instruments may lead to variations in the spectral features captured and, consequently, their modeling performance for selenium quantification.

This study presents a pioneering, systematic comparison of three NIR spectrometer types (benchtop FT-NIR, portable Micro NIR 1700, and handheld Micro PHAZIR RX) for the rapid quantification and classification of selenium in Pacific oysters (*Crassostrea gigas*). This work represents the first comprehensive evaluation of multiple NIR instrument platforms for selenium analysis in oysters, facilitating the assessment of NIR technology across laboratory and field-deployable settings in aquaculture. We hypothesize that the predictive accuracy of portable spectrometers will be comparable to that of benchtop systems, validating their potential for rapid on-site screening. Accordingly, this study aims to: (1) develop robust quantitative models for selenium prediction; (2) construct effective qualitative models for selenium level discrimination; and (3) critically evaluate the applicability and limitations of each instrument platform. This comprehensive evaluation is anticipated to establish a robust methodological foundation for the rapid, non-destructive quality control of selenium-enriched aquatic products.

## 2. Materials and Methods

### 2.1. Experimental Materials

Pacific oysters were obtained from the Kongtong Island Genetic Breeding Base in Yantai, Shandong Province, China. The study was conducted under controlled aquaculture conditions, with diploid, triploid, and tetraploid strains being reared in separate 6 m^3^ recirculating tank systems to prevent cross-contamination and allow for independent environmental management. A total of 600 oysters (200 each of the three ploidy strains) were acclimatized for 7 days prior to experimentation. Water temperature in each tank was maintained at 15.0 ± 0.5 °C with continuous aeration to ensure adequate oxygenation. All specimens exhibited intact shells, vigorous activity, and consistent morphological characteristics: shell height 7–12 cm, shell length 4–7 cm, shell width 3–5 cm, and total weight 90–160 g. A 15-day selenium enrichment trial was conducted in December 2023 using commercial selenium yeast (Angel Yeast Co., Ltd., Yichang, China; selenium content: 2000 mg/kg) as the dietary selenium source. Throughout this period, the health status of the oysters was monitored daily within each tank, with observations focused on shell closure response and general activity levels, in addition to the daily random sampling. A total of 161 valid samples (56 diploid, 50 triploids, 55 tetraploid) were collected through this sampling regimen for subsequent analysis.

### 2.2. Sample Preparation

After shucking the fresh oysters, the soft tissues were thoroughly removed. The tissues taken from every three oysters were combined and homogenized into a slurry. The homogenate was divided into two portions and stored in transparent plastic bags: one portion was allocated for NIR spectral analysis, while the other was designated for chemical determination of selenium content. All samples were stored at −80 °C. The fresh oyster homogenate was lyophilized for 48 h using a freeze-dryer and then thoroughly ground into a fine powder using a mortar and pestle. The resulting powder was stored at −20 °C. The spectra of the fresh tissue homogenate were collected using the portable Micro NIR 1700 spectrometer (JDSU, Milpitas, CA, USA) and the handheld Micro PHAZIR RX spectrometer (Thermo Fisher, Waltham, MA, USA). The spectra of the freeze-dried powder samples were acquired using the Antaris II Fourier Transform Near-Infrared (FT-NIR) spectrometer (Thermo Fisher, Waltham, MA, USA).

### 2.3. Determination of Selenium Content

The selenium content in the oysters was determined according to the Third Method (Hydride Generation-Atomic Fluorescence Spectrometry, HG-AFS) specified in the Chinese National Food Safety Standard GB 5009.93-2017, “National Food Safety Standard—Determination of Selenium in Foods [28].”

### 2.4. Portable Near-Infrared Spectral Acquisition

**Figure 1:**
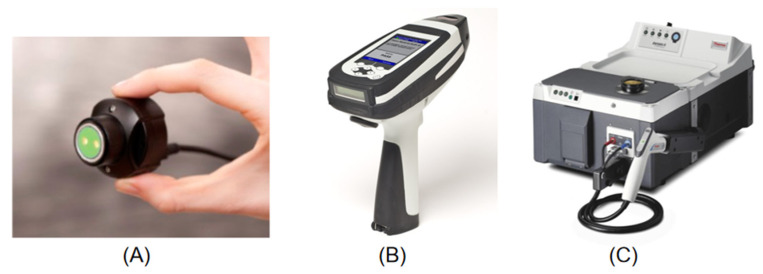
Schematic diagram of the three NIR spectrometers: (**A**) Micro NIR 1700; (**B**) Micro PHAZIR RX; and (**C**) FT-NIR spectrometer.

To compare the performance of near-infrared spectrometers, three instruments were used in this study: the Micro NIR 1700 (MN), the Micro PHAZIR RX (MP), and the Fourier Transform Near-Infrared spectrometer (FT-NIR, FN), as shown in Figure 1.

The MN is a micro-system that incorporates a Linear Variable Filter (LVF) monochromator, along with a light source, light-collecting components, electronics, and basic operating software. Its spectral acquisition range is 908–1676 nm, covering the absorption bands of high-energy chemical bonds in organic matter. The instrument has a scanning speed of 4 scans per second and dimensions of only 45 × 42 mm, making it one of the smallest NIR spectrometers on the market. The MN processes the light source via a beam splitter, illuminates the sample using a detector, and ultimately obtains an NIR spectrum as a function of wavelength. During spectral acquisition, the probe temperature was maintained between 40 and 50 °C, and each sample was scanned 10 times. Spectral data analysis was performed using The Unscrambler X 10.4 software, where the spectra for each oyster sample were averaged.

The MP is the world’s first handheld NIR analysis device, utilizing a grating light modulator technology. This micro NIR spectrometer operates independently without requiring external devices, enabling truly portable NIR analysis. It acquires spectra in the wavelength range of 1624–2460 nm, covering the overtone and combination bands of covalent bonds. However, its resolution is relatively low at 22 nm. Before each spectral acquisition, the instrument required a 10 min warm-up period and a self-check. Prior to spectral collection, the built-in program was used to set the device to spectral acquisition mode to eliminate background interference. Each sample was scanned 5 times, and the best spectrum for each sample was selected for subsequent analysis using the instrument’s accompanying Method Generator software (Version 5.3.0.42).

Prior to scanning with the FN spectrometer, the RESULT integration software was used to create a workflow for spectral acquisition. The spectrometer was powered on and warmed up for at least 0.5 h. Oyster powder samples were placed in a quartz cup with a diameter of 1 cm, filled to a height of 1.5 cm. Spectra were acquired using diffuse reflectance mode in the wavenumber range of 10,000–4000 cm^−1^ (wavelength 1000–2500 nm). Each measurement consisted of 32 scans at a resolution of 8 cm^−1^, and the absorption spectra were expressed as log(1/R) (diffuse reflectance). A background spectrum was collected before each sample measurement to eliminate background interference, with a single measurement time controlled within 1 min.

### 2.5. Spectral Data Preprocessing

Spectral preprocessing methods were applied prior to model construction to minimize the effects of external environmental factors, sample density, and temperature variations on the spectral data, as well as to remove high-frequency random noise, baseline drift, and issues related to sample inhomogeneity.

For the MP instrument, the preprocessing methods tested included the original spectrum (SP), Savitzky–Golay (SG) filtering, Multiplicative Scatter Correction (MSC), and Standard Normal Variate (SNV). For the MN and FN instruments, the methods included the original spectrum (SP), First Derivative (FD), Second Derivative (SD), SG filtering, and Norris Derivative Filtering (NDF). These techniques were used in combination to reduce noise while preserving latent spectral data related to chemical information, thereby identifying the most suitable preprocessing method. Furthermore, the Mahalanobis Distance (MD) was used to identify and remove outlier samples from the models.

Based on the selected preprocessing methods, quantitative models for the selenium content in Pacific oysters were established using the respective instruments. Qualitative models were also constructed to categorize selenium content into different ranges. To optimize the NIR data across the entire wavelength range, the aforementioned spectral preprocessing methods were applied sequentially.

### 2.6. Quantitative Model Construction and Validation

A total of 161 NIR spectra collected from Pacific oysters were randomly divided into a calibration set and a validation set. Partial Least Squares (PLS) regression was employed for the quantitative analysis of selenium content. The full wavelength range acquired by each instrument was used for modeling. Model optimization and validation were performed to enhance prediction accuracy and robustness. To achieve the best modeling performance, various spectral preprocessing methods were screened and compared, and the combination yielding the optimal statistical indicators was selected to establish the final model.

Prior to modeling, outliers were detected and removed. Initially, the MD was used to identify and remove outlier sample points. Subsequently, Dixon’s test was applied to further exclude abnormal spectra, thereby enhancing model stability and robustness. To mitigate the risk of overfitting, a cross-validation strategy was adopted during model development: three samples were randomly selected from the calibration set as the validation set each time, with the remaining samples used for modeling. This process was repeated until all samples had been validated once and only once, yielding a cross-validated prediction value for each sample. External validation was performed using an independent sample set, separate from the calibration set, to test the model’s generalizability. To ensure reliability and representativeness, the validation set comprised approximately 1/9 of the total samples.

Model performance was evaluated using the following statistical parameters: Root Mean Square Error of Calibration (RMSE_C_), Correlation Coefficient of Calibration (R_C_), Root Mean Square Error of Cross-Validation (RMSE_CV_), Correlation Coefficient of Cross-Validation (R_CV_), Root Mean Square Error of Prediction (RMSE_P_), Correlation Coefficient of Prediction (R_P_), and the Ratio of Performance to Deviation (RPD). A high-quality model is typically characterized by a high correlation coefficient (R^2^ close to 1) and low values for RMSE_CV_ and RMSE_P_, indicating strong predictive ability. A larger RPD value indicates better quantitative predictive performance of the model. RPD < 1.4 indicates an unreliable model, 1.4 ≤ RPD < 2.0 suggests the model has some reference value and can be used for approximate prediction, RPD ≥ 2.0 indicates the model has high reliability and quantitative accuracy [29,30].

The relevant formulas for these parameters are as follows:

(1) $$RMSE=\sqrt{\frac{1}{n}\sum_{i=1}^{n}{\left(y_{i}-\hat{y_{i}}\right)}^{2}}$$

(2) $$R^{2}=1-\frac{\sum_{i=1}^{n}{\left(y_{i}-\hat{y_{i}}\right)}^{2}}{\sum_{i=1}^{n}{\left(y_{i}-\bar{y}\right)}^{2}}$$

(3) $$RPD=\frac{SD}{RMSE}$$

where y_i_ is the measured value of the i-th sample, $\hat{y_{i}}$ is the predicted value from the model, $\bar{y}$ is the mean of the measured values, SD is the standard deviation of the measured values, and n is the number of samples.

### 2.7. Construction and Validation of Qualitative Models

Approximately 50 samples from each of the three instruments were selected to establish qualitative models for distinguishing oyster samples according to different concentration ranges of selenium. The developed models were designed to effectively identify and classify samples into distinct selenium content intervals. The construction of the qualitative models was based on the principles of chemometric analysis, employing Discriminant Analysis (DA) in combination with the MD for sample discrimination at a 95% confidence level. Spectral data processing and model development were carried out using TQ Analyst software (Version 9.8.208).

For the qualitative models developed with the MN and FN instruments, the Performance Index (PI) and the Cumulative Contribution Rate (CCR) were used as evaluation metrics. A higher PI value generally indicates a stronger discriminative ability of the model for sample categories, along with better stability and reliability. The maximum value of PI is 100, and values closer to 100 represent superior model performance. The CCR is one of the most important indicators in Principal Component Analysis (PCA), used to determine how much of the total data variance is explained by the first k principal components. A higher cumulative contribution rate indicates that the first k principal components capture more of the original information [31]. The formulas are as follows:

(4) $$PI=\frac{\left|\mathrm{actual}-\mathrm{calculated}\right|}{\mathrm{expected}}\times 100$$

(5) $$CCR=\frac{\sum_{i=1}^{k}\lambda_{i}}{\sum_{i=1}^{p}\lambda_{i}}\times 100\%$$

where λ_i_ is the eigenvalue of the i-th principal component, p is the total number of principal components, and k is the number of principal components included in the cumulative sum.

For the MP instrument, the Method Generator software (Version 5.3.0.42) was used to process the acquired spectral data. The qualitative identification of samples was achieved by using the “Classify” function within the software. The models were validated using independent samples, and their reliability was assessed primarily based on the discrimination accuracy rate. The evaluation metric used was the Area Under the Receiver Operating Characteristic Curve (AUC). A larger AUC value indicates a stronger discriminative ability of the model, with values closer to 1 signifying higher predictive accuracy [32].

## 3. Results and Discussion

### 3.1. Descriptive Statistics of Selenium Content Indicators

**Figure 2:**
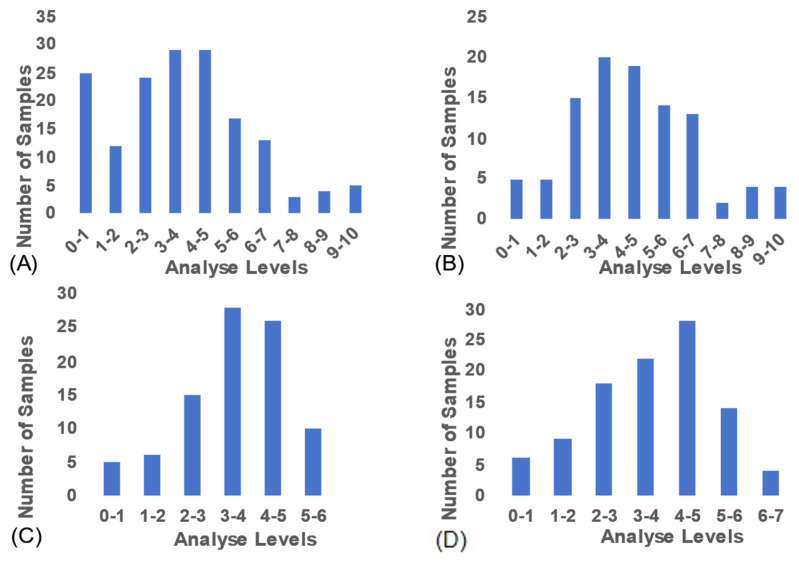
(**A**) Distribution of selenium content across all samples. (**B**–**D**) Selenium content distribution of samples analyzed by the MP, MN, and FN instruments, respectively.

The selenium content in the Pacific oyster samples used in this study exhibited a wide distribution range (Figure 2A), a prerequisite for establishing reliable NIR quantitative analysis models. The distribution profiles for the subsets analyzed by the Micro MP, MN, and FN instruments are shown in Figure 2B–D, respectively.

After removing outliers and performing validation processing, quantitative models for selenium content in oysters of different ploidies were established based on the NIR spectra collected by the different instruments. The sample information for each model in the calibration and validation sets is shown in Table 1. Due to limitations imposed by the proprietary software of the MP instrument, external validation of its data could not be conducted.

For the quantitative model developed using the MP instrument, the selenium content of the modeling samples averaged 4.440 ± 2.106 mg/kg, with values ranging from 0.458 to 10.000 mg/kg. For the MN instrument, the average selenium concentration was 3.515 ± 1.213 mg/kg, with a range of 0.730 to 5.523 mg/kg. In the model established using the FN instrument, the samples exhibited a mean selenium content of 3.641 ± 1.490 mg/kg, ranging from 0.438 to 6.945 mg/kg.

The selenium concentrations measured in this study fall within the range reported for other bivalve mollusks. For instance, mean selenium levels of 3.34 ± 0.96 mg/kg and 2.79 ± 0.89 mg/kg were reported for *S. palmula* and *C. corteziensis* from the southeastern Gulf of California, while farmed Mediterranean mussels (*Mytilus galloprovincialis*) from Türkiye and Bulgaria exhibited concentrations ranging from approximately 1.305 to 1.957 mg/kg [33,34]. The comparability of our data to these literature values supports the environmental relevance of the samples used. It is important to clarify that the relatively wide concentration range was intentionally achieved through a controlled selenium enrichment experiment. This design was essential to obtain a sufficient spread for developing robust NIR calibration models. Therefore, the elevated selenium levels in some samples result from experimental intervention and do not represent natural background conditions.

### 3.2. Spectral Characteristics Analysis of the Three Instruments

**Figure 3:**
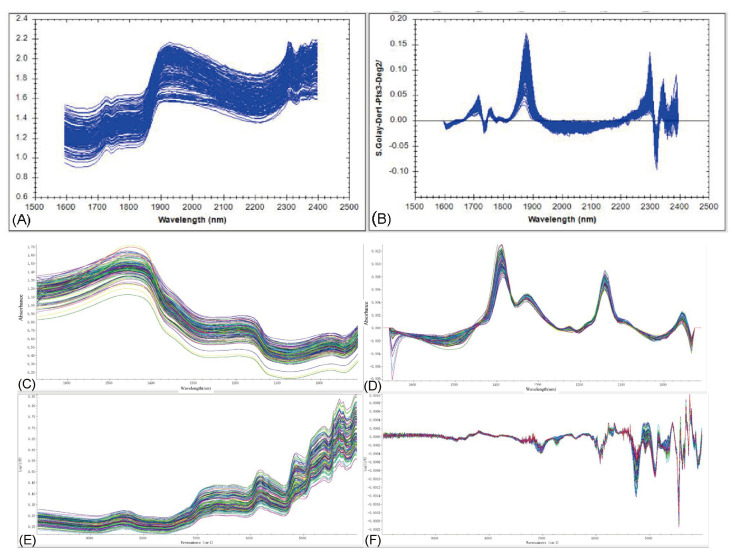
Panels (**A**,**C**,**E**) show the raw spectra acquired using the MP, MN, and FN instruments, respectively, while panels (**B**,**D**,**F**) present the corresponding first-derivative spectra.

Figure 3 displays the raw and preprocessed NIR spectra of all oyster samples collected using the different instruments. Distinct absorption features were observed for each instrument after preprocessing.

The spectra obtained with the MP instrument exhibited significant absorption peaks in the 1800–2050 nm and 2200–2350 nm regions. The 1800–2050 nm region is primarily attributed to the strong combination bands of O–H bonds, with signals originating from both bound water and protein-water interactions. The 2200–2350 nm region corresponds to combination bands of C–H bonds, mainly associated with vibrations in protein side chains and lipid structures. Information from these bands can reflect the moisture status, protein conformation, and lipid composition of the samples.

The preprocessed spectra acquired with the MN instrument showed apparent absorption characteristics in the 1000–1200 nm and 1300–1500 nm regions. The former corresponds primarily to the first overtone absorption bands of C–H, O–H, or C–O stretching vibrations, reflecting the characteristics of organic compounds containing hydroxyl or carboxyl groups. The latter is related to combination bands involving N–H stretching and bending vibrations, characterizing the absorption features of proteins and other nitrogen-containing functional groups.

The spectra measured with the FN instrument displayed distinct absorption features in the regions of 4200–5500 cm^−1^ (1820–2380 nm) and 5800–7200 cm^−1^ (1390–1720 nm). The 4200–5500 cm^−1^ region is mainly contributed to by combination vibration bands of C–H bonds, reflecting characteristics of protein side chains and lipid structures, while also containing absorption information from bound water and combination vibrations of N–H and O–H bonds. The 5800–7200 cm^−1^ region corresponds predominantly to the first overtone bands of N–H and O–H stretching vibrations, which are closely related to protein structure and sample moisture status. These absorption characteristics reveal the sensitivity of FN spectra to the hydrogen-bonding environment and organic molecular structure within the samples, providing a foundation for subsequent model development.

### 3.3. Development and Optimization of Quantitative Models

#### 3.3.1. Spectral Analysis

To develop optimal quantitative models for predicting selenium content in Pacific oysters, various spectral preprocessing methods and their combinations were compared and optimized. The workflow involved initially preprocessing the entire spectral range, followed by selecting the optimal modeling wavelengths based on characteristic absorption peaks within different regions. Quantitative models were then established under different numbers of principal components, and their performance was evaluated using metrics such as the Root Mean Square Error of Prediction (RMSE_P_) and the Coefficient of Determination (R^2^). The model associated with the highest R^2^ and the lowest prediction error was selected as corresponding to the best preprocessing method. Detailed information on the preprocessing methods, the number of principal components, and the selected wavelength ranges for the quantitative models of different instruments is summarized in Table 2.

#### 3.3.2. Quantitative Model Performance Evaluation and Analysis

**Figure 4:**
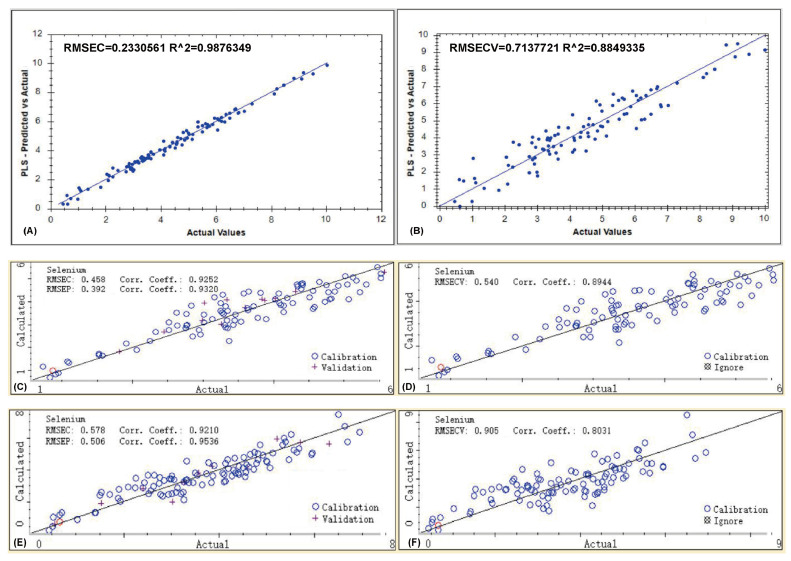
Performance comparison of selenium content prediction models based on three NIR instruments. (**A**,**B**) Results for the MP instrument; (**C**,**D**) Results for the MN instrument; (**E**,**F**) Results for the FN instrument.

Quantitative models for the selenium content in Pacific oysters were established using three different near-infrared spectrometers. After determining the optimal preprocessing methods, three quantitative models were developed using the MP, MN, and FN instruments (Figure 4). The model performance was comprehensively evaluated using multiple statistical indicators from the calibration, cross-validation, and external validation stages, including the Root Mean Square Error (RMSE_C_, RMSE_CV_, RMSE_P_), the Determination Coefficient (R_C_, R_CV_, R_P_), and the Residual Predictive Deviation (RPD_CV_, RPD_P_). The specific values for each instrument are listed in Table 3.

The model based on the MP spectrometer demonstrated the highest fit within the calibration set, with an RMSE_C_ of 0.233 and an R_C_ of 0.988. However, during cross-validation, the performance declined, with the RMSE_CV_ rising to 0.713 and the R_CV_ dropping to 0.885. The notable disparity between R_C_ and R_CV_ indicates a potential tendency of overfitting. Although the RPD_CV_ remained high at 2.95, suggesting good quantitative potential, the model’s generalization stability is inferior to that of the MN instrument. External validation was not feasible due to limitations imposed by the instrument’s proprietary software. The MP instrument operates in the 1624–2460 nm range, which is more prone to absorption from water and the sample matrix background, a phenomenon that often results in models with excellent fit on the training set but compromised generalization ability, as also mentioned in reviews of handheld NIR applications [35].

For the model developed using the MN instrument, the calibration set showed an RMSE_C_ of 0.458 and an R_C_ of 0.925, indicating good fitting precision during the training phase. In cross-validation, the model achieved an RMSE_CV_ of 0.540, an R_CV_ of 0.894, and an RPD_CV_ of 2.23, demonstrating high internal stability and good predictive capability. During external validation, the model performed robustly with an RMSE_P_ of 0.392, an Rp of 0.932, and an RPD_P_ of 2.46, all of which exceed the generally accepted threshold for quantitative prediction (RPD > 2.0) [29,30]. This excellent robustness can be attributed to the instrument’s spectral range (908–1676 nm), which primarily covers the overtone and combination absorption bands of bonds such as C-H and N-H. This range effectively reflects the structural features of selenium-binding proteins while being less affected by the strong absorption interference from O-H bonds in water. Previous studies have also noted that the short-wave NIR region often provides better calibration stability for aqueous biological samples, which is consistent with the performance observed in this study [36].

The model established with the FN instrument showed a calibration RMSE_C_ of 0.578 and an R_C_ of 0.921, indicating a slightly lower fitting accuracy compared to the other two models. In cross-validation, the performance was relatively poor with an RMSE_CV_ of 0.905, an R_CV_ of 0.803, and an RPD_CV_ of 1.65, suggesting weaker internal consistency and predictive stability. Interestingly, the model achieved the best performance in external validation, with an RMSE_P_ of 0.506, an R_P_ of 0.954, and an RPD_P_ of 2.60. This superior external predictive ability can be attributed to the use of powdered samples. Powdering effectively reduces the spatial heterogeneity within samples caused by variations in particle size, tissue structure, and local moisture distribution [37,38]. This process significantly enhances the stability and reproducibility of the spectral signals and minimizes water interference. Consequently, the model demonstrates excellent generalization capability when applied to the independent validation set, highlighting its suitability for high-precision quantitative analysis under laboratory conditions.

In summary, the performance of the models varied among the three instruments. The MP instrument achieved the highest linearity in the calibration set (R_C_ = 0.988) but showed a clear decline in R_CV_ during cross-validation, suggesting a potential overfitting tendency. The MN instrument maintained good consistency across the calibration, validation, and prediction stages (RPD_P_ = 2.46), demonstrating strong robustness and generalization ability, making it the most balanced performer overall. Although the FN instrument showed relatively ordinary performance in the calibration and cross-validation stages, its superior external predictive ability (R_P_ = 0.954, RPD_P_ = 2.60) is attributed to the diminished spectral interference from water and tissue structure in powdered samples, making it more appropriate for precise quantitative analysis of powdered samples in a laboratory setting.

The successful development of these quantitative PLS models fundamentally relies on the sensitivity of NIR spectroscopy to the molecular environment of selenium-binding proteins. The incorporation of selenium as selenocysteine alters protein conformation and hydrogen-bonding networks, leading to measurable spectral shifts [39,40]. This principle is supported by studies showing discernible infrared spectral changes in selenium-containing amino acids and successful quantitative analysis of selenium in dairy matrices using infrared spectroscopy [41,42]. These findings collectively validate that the spectral variations captured by our NIR instruments are intrinsically linked to selenium content, enabling the quantitative predictions demonstrated here.

Overall, this study successfully achieved rapid quantitative prediction of selenium content in Pacific oysters using three different NIR spectrometers, proving the feasibility and application potential of NIR technology for the analysis of trace elements in shellfish. Considering the model fitting accuracy, stability, and external prediction results collectively, the model established with the MN instrument performed the most robustly and can be considered the preferred solution for routine rapid testing. Meanwhile, the FN instrument, under powdered sample conditions, demonstrated superior prediction accuracy, making it more suitable for high-precision quantitative requirements in laboratory environments.

### 3.4. Development of Qualitative Discrimination Models

To enable rapid discrimination of selenium enrichment status in marine environments, we classified the selenium content in *Crassostrea gigas* samples into three levels: 0–1 mg/kg, 1–3 mg/kg, and >3 mg/kg. This classification scheme comprehensively considers nutritional relevance, environmental enrichment characteristics, and potential ecological risk. Specifically, a selenium concentration of 0–1 mg/kg was regarded as the baseline or nutritional background level; a concentration of 1–3 mg/kg reflected significant bioaccumulation; and a concentration exceeding 3 mg/kg indicated a high enrichment level, denoting potential ecological or food safety risks.

This classification is consistent with selenium concentration ranges reported in previous studies on seafood and bivalve mollusks, thereby providing a meaningful framework from both environmental and food safety perspectives. For instance, earlier studies reported that selenium concentrations in certain marine products, such as fish, typically range from 0.12 to 1.27 mg/kg. In contrast, bivalves like mussels and oysters exhibit greater variability, with reported selenium contents ranging from 0.28 to 0.53 mg/kg to higher levels of 0.5–3.6 mg/kg. These findings indicate substantial differences in selenium accumulation among marine organisms, reflecting the coexistence of multiple enrichment states and heterogeneous distribution patterns [34,43,44]. The established scheme, supported by literature evidence, provides a rational basis for developing qualitative discrimination models.

Based on this classification scheme, qualitative discrimination models were established, with the correct classification rate adopted as the primary performance evaluation metric. In this study, qualitative identification models were developed using three different near-infrared spectrometers, and the corresponding spectral preprocessing methods and model performance parameters are summarized in Table 4.

#### 3.4.1. Classification Model Based on the MP Instrument

**Figure 5:**
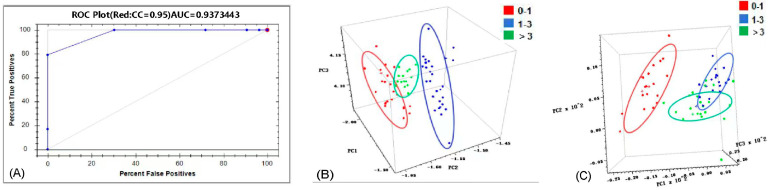
(**A**) ROC curve of the MP instrument. (**B**) PCA score plot of the MN instrument. (**C**) PCA score plot of the FN instrument.

The spectra acquired using the MP instrument were qualitatively analyzed within the “Classify” module of the Method Generator software. As shown in Table 4, the model achieved a correct recognition rate of 100%, indicating its effectiveness in distinguishing oyster samples with varying selenium content levels. Figure 5A shows the Receiver Operating Characteristic (ROC) curve for the MP instrument. The ROC curve depicts the relationship between sensitivity and specificity (1-specificity). A curve closer to the top-left corner and a larger Area Under the Curve (AUC) indicate a stronger discriminative ability of the model. An AUC value closer to 1 signifies higher predictive accuracy. Model performance is generally considered acceptable when AUC > 0.7 [45]. The AUC value of this model was 0.937, approaching 1, which further confirms its high discriminative accuracy in classifying oyster samples into different selenium concentration ranges.

#### 3.4.2. PCA Discrimination Models Based on the MN and FN Instruments

The spectra collected by the MN and FN instruments were analyzed using TQ software. Qualitative discrimination models were established using Principal Component Analysis (PCA). The results indicated that the models could correctly distinguish samples based on different selenium content levels (Table 4). The model for the MN instrument was established using 5 principal components, achieving a cumulative contribution rate (CCR) exceeding 98.61% and a Performance Index (PI) of 89.3. The model for the FN instrument used 9 principal components, with a CCR of 97.59% and a PI of 90.2, also achieving accurate discrimination among the three classes. As shown in the principal component score plots in Figure 5B,C, the score clusters representing samples with different selenium levels are clearly separated in the space defined by the first three principal components (PC1, PC2, PC3). The minimal overlap of the 95% confidence interval ellipses visually demonstrates the effectiveness of combining NIR spectroscopy with PCA for selenium content level identification.

#### 3.4.3. Performance Evaluation and Discussion of Qualitative Models

The classification model developed using the MP instrument demonstrated excellent performance in discriminating selenium content levels (0–1, 1–3, and >3 mg/kg) in Pacific oysters, achieving a correct recognition rate of 100% and an AUC value of 0.937. Meanwhile, the discrimination models established using the MN and FN instruments combined with Principal Component Analysis (PCA) also effectively distinguished samples with different selenium levels. These results indicate that even without employing complex classification algorithms such as Support Vector Machines (SVM) or neural networks, discriminant features related to selenium accumulation status can be effectively extracted from NIR spectra using basic chemometric methods such as PCA.

The high classification accuracy of our models can be attributed to the distinct spectral patterns induced by different levels of selenium accumulation. The substitution of sulfur by selenium in amino acids significantly affects molecular vibrations, creating spectral fingerprints that are distinguishable by chemometric methods [39,40]. The fact that selenocysteine can be specifically identified by optical probes further confirms the unique spectroscopic signature of selenium-containing structures [46]. Therefore, the observed clear separation between selenium content classes in our PCA and classification models is a direct reflection of these underlying molecular-level alterations.

This finding is consistent with conclusions from previous studies. For instance, a recent study successfully distinguished cadmium-contaminated mussels from normal samples using NIR reflectance spectroscopy with an accuracy exceeding 99.9%, further confirming the feasibility of NIR spectroscopy combined with chemometrics for the rapid screening of heavy metal pollution in shellfish [47]. Additionally, studies on the classification of heavy metal pollution risk levels in soil and environmental samples using Vis-NIR or NIR spectroscopy have reported overall classification accuracies generally ranging between 75% and 100%, further supporting the reliability of the method used in this study [48].

From a mechanistic perspective, the accumulation of selenium within organisms often induces changes in protein conformation, the forms of organ selenium compounds, or tissue microstructures. These changes can correspondingly alter the vibrational characteristics of chemical bonds such as C-H, N-H, and O-H. NIR spectroscopy is highly sensitive to the overtone and combination bands of these chemical bonds, thereby enabling the capture of spectral information related to selenium content, which provides a basis for chemometric classification. Indeed, the widespread successful application of NIR technology in quality assessment and pollution discrimination of complex matrices, foods, and biological samples is fundamentally based on its high responsiveness to molecular vibrational characteristics [49].

In terms of practical application, different instrument systems possess distinct advantages: the MP instrument, with its exceptional classification accuracy, simple operation, and portability, is particularly suitable for rapid on-site screening and preliminary monitoring. In contrast, although the MN and FN instruments require certain spectral preprocessing and PCA modeling steps, they offer greater versatility and lower dependency on the physical state of the sample, making them more suitable for batch sample processing or systematic monitoring tasks in laboratory settings.

This study also has several limitations. Firstly, the limited sample size and relatively singular sources (factors such as geography, season, and growth environment were not fully covered) may affect the model’s generalizability. Secondly, the three predefined selenium content levels may not fully reflect the continuous distribution characteristics of selenium concentration in actual environments; future studies could consider introducing more gradations to enhance applicability. Furthermore, NIR spectra are prone to interference from factors such as sample moisture and tissue status. It is recommended that future research incorporate more robust preprocessing and standardization strategies to further enhance model stability and applicability.

In summary, this study validates the effectiveness of combining NIR spectroscopy with chemometrics for discriminating selenium content levels in shellfish (Pacific oysters). It provides a non-destructive, efficient, and low-cost technical approach for the rapid screening of heavy metal pollution in marine environments and for monitoring the safety and quality of aquatic products.

## 4. Conclusions

This study successfully established and validated NIR spectroscopy as a rapid, non-destructive technique for assessing selenium content in Pacific oysters. Comparative evaluation of three spectrometer types (benchtop FT-NIR, portable Micro NIR 1700, and handheld Micro PHAZIR RX) confirmed that all are suitable for developing robust analytical models. Specifically, a quantitative PLS model based on the portable Micro NIR 1700 demonstrated high accuracy and generalization capability, while qualitative discrimination models across all instruments effectively classified oysters into different selenium concentration ranges with excellent accuracy. The findings underscore the critical influence of sample morphology on model performance. Collectively, this work provides a practical methodological foundation for the rapid, on-site nutritional evaluation and quality control of selenium-enriched aquatic products.

## Figures and Tables

**Table 1:** Composition of selenium content in the calibration and validation sets.

Instrument	Calibration Set			Validation Set		
	Average	Range	N	Average	Range	N *
Micro PHAZIR RX(MP)	4.440 ± 2.106	0.458~10.00	101	-	-	-
Micro NIR 1700(MN)	3.515 ± 1.213	0.730~5.523	90	3.507 ± 0.953	1.810~5.567	12
FT-NIR(FN)	3.641 ± 1.490	0.438~6.945	101	3.640 ± 1.756	0.554~6.310	11

* Represents the number of samples.

**Table 2:** Preprocessing methods, number of principal components, and selected wavelength.

Instrument	Preprocessing *	Number of PCs	Wavelength Range
Micro PHAZIR RX(MP)	SNV, MSC	19	1800–2300 nm
Micro NIR 1700(MN)	FD, SG (7, 3)	9	1106–1251 nm, 1348–1550 nm
FT-NIR(FN)	FD, SG (7, 5)	10	1390–1720 nm, 1820–2380 nm

* Represents the preprocessing method used by the model. SNV: Standard normal variate, MSC: Multiplicative scatter correction, SG: Savitzky–Golay filter, FD: First derivative.

**Table 3:** Performance indicators of the quantitative models developed with different instruments.

			Validation Set					
Instrument	Calibration Set		Cross-Validation			External Validation		
	RMSE_C_	R_C_	RMSE_CV_	R_CV_	RPD_CV_	RMSE_P_	R_P_	RPD_EV_
Micro PHAZIR RX (MP)	0.233	0.988	0.713	0.885	2.95	-	-	-
Micro NIR 1700 (MN)	0.458	0.925	0.540	0.894	2.23	0.392	0.932	2.46
FT-NIR (FN)	0.578	0.921	0.905	0.803	1.65	0.506	0.954	2.60

**Table 4:** Preprocessing methods and performance parameters of the qualitative models for different instruments.

**Instrument**	**Preprocessing**	**Correct Recognition Rate**	**AUC** ^1^	**Accurate Discrimination**
Micro PHAZIR RX	SG (7, 2), MSC	100%	0.937	Yes
**Instrument**	**Preprocessing**	**Number of PCs/(CCR** ^2^**)**	**PI** ^3^	**Accurate Discrimination**
Micro NIR 1700	SP, MSC	5/(98.61%)	89.3	Yes
FT-NIR	FD, SG (7, 5)	9/(97.59%)	90.2	Yes

^1^ Area Under Curve, ^2^ Cumulative Contribution Rate, ^3^ Performance Index.

## Data Availability

The original contributions of this study are presented in this article. Further inquiries can be directed to the corresponding author.

## Abbreviations

The following abbreviations are used in this manuscript:

NIR	Near-infrared
PLS	Partial least squares
AUC	Area Under Curve
MP	Micro PHAZIR RX
MN	Micro NIR 1700
FN	FT-NIR
MD	Mahalanobis Distance
CCR	Cumulative Contribution Rate
PI	Performance Index
PCA	Principal Component Analysis
